# A Scientific Proposal for Surgical Decision-Making in Occult Intertrochanteric Fractures Based on Finite Element Analysis

**DOI:** 10.7759/cureus.44491

**Published:** 2023-08-31

**Authors:** Mitsuaki Noda, Kazuhiko Adachi, Shunsuke Takahara

**Affiliations:** 1 Orthopaedics, Nishi Hospital, Kobe, JPN; 2 Department of Mechanical Engineering, Chubu University, Kasugai, JPN; 3 Department of Orthopaedics, Hyogo Prefectural Kakogawa Medical Center, Kakogawa, JPN

**Keywords:** yield stress, occult fracture, weight-bearing restrictions, fracture extension, stress analysis, finite element method, femoral intertrochanteric fractures

## Abstract

Background

In the treatment of femoral intertrochanteric fractures, there is still a lack of consensus on the optimal approach for isolated greater trochanteric fractures and insufficient intertrochanteric fractures. The limited number of patients and restricted access to accurate assessment of fracture extension using magnetic resonance imaging contribute to the unclear treatment strategy. This study aims to utilize finite element (FE) analysis to analyze stress values at the fracture line and investigate their influence on intertrochanteric fracture extension under different loading conditions. The hypothesis is that fracture extension occurs following certain conditions, supporting the need for surgery based on scientific evidence.

Methodology

Osseous data from a computed tomography (CT) scan was used to create a proximal femur FE model using FEA software. CT scan data were converted to Digital Imaging and Communications in Medicine format and used to generate the FE model. Trabecular bone and cortex were meshed into tetrahedral elements. The model consisted of 1,592,642 elements and 282,530 nodes. Two models were created, namely, healthy proximal femur (HF) and femoral insufficient intertrochanteric fracture (FIF). Material properties were assigned based on CT values and conversion equations. The distal end of the femur was constrained. Stress analysis using the dynamic explicit approach was performed. Von Mises stresses were calculated for the proximal femur. The number of elements exceeding yield stress was counted to predict fracture risk by focusing on fracture line spots. In this study, the distribution of von Mises stress was compared between the HF and the FIF models. Six loading combinations were considered, namely, two weight-bearing conditions (3 W loading simulating for walking and 1/3 W for touch-down standing) and three hip flexion angles (0°, 15°, and 23°).

Results

Under 3 W loading, no significant stress elevations were observed in the HF model at any flexion angles. However, the FIF model exhibited increased stress at the site of the posterior fracture line extension. This stress-induced element destruction was observed in both cortical and cancellous bone. For the 1/3 W loading condition, only minimal stress elevation was observed in both HF and FIF models. To assess the influence on fracture extension, the number of yielded elements was evaluated along the fracture line edges (greater trochanter and middle of the intertrochanteric ridge). Under 3 W loading, the HF model had only one yielded element, indicating minimal fracture risk. In contrast, the FIF model exhibited a notable presence of yield elements in various regions (total/greater trochanter/shaft) at different flexion angles: 0° (115/16/28), 15° (265/158/23), and 23° (446/233/34). Under the 1/3 W loading condition, neither the HF nor the FIF models showed any yielding elements, regardless of the direction of external force.

Conclusions

The results demonstrated elevated stress levels at the fracture line in the FIF model, particularly during walking, indicating a higher risk of fracture extension at the flex position. However, under reduced weight-bearing conditions, the stress at the fracture site remained within the yield stress range, suggesting a relatively low risk of fracture extension. These findings hold significant clinical implications for developing surgical protocols that consider patients’ compliance with weight-bearing restrictions.

## Introduction

The incidence of femoral intertrochanteric fractures among elderly patients is globally increasing [1]. Treatment protocols for these fractures have already been established in most cases [2,3]. Among them, isolated greater trochanteric fractures and insufficient intertrochanteric fractures are considered rare intertrochanteric fractures. These two fractures are closely combined, and magnetic resonance imaging (MRI) has been used to depict the fracture line extending from the greater trochanter to the medial femoral cortex along the intertrochanteric ridge [4].

Nevertheless, there is a lack of consensus on the optimal treatment approach for this insufficient fracture [4]. Some studies suggest that surgery should be performed regardless of the extent of the fracture line, except in the case of medical contraindications [5]. On the other hand, the widely accepted opinion is that surgery should be recommended when the fracture line extends through more than 50% of the longitudinal axis [6]. Alam et al. [7] reported positive outcomes from conservative treatment of incomplete intertrochanteric fractures, even when the fracture line reached the intertrochanteric area. Therefore, the lack of uniformity in the treatment strategy can be attributed to a smaller number of patients with this fracture in the published literature and the limited access to MRI for accurate assessment of fracture extension. Despite the failure of clinical studies to provide a treatment protocol, to our knowledge, there are no studies specifically focussing on this issue.

With recent advancements in scientific technology, finite element methods (FEMs) offer specific advantages in predicting mechanical stress at designated regions with accurate reproduction of complex geometry and consideration of different material properties under various conditions. Finite element (FE) analysis can foresee the risk of femoral fractures more accurately than bone mineral density analysis [8]. Additionally, FE simulation allows for the investigation of crack propagation and fracture toughness using knowledge of the elastic properties [9,10]. This study aims to analyze the stress values at the fracture line that influence the extension of intertrochanteric fractures under different loading conditions. We hypothesize that further fracture extension is inevitable after a certain amount of weight-bearing and hip joint angles, thus suggesting surgery, as indicated by scientific results.

## Materials and methods

Finite element modeling

We obtained a sample osseous data from a computed tomography (CT) scan of an anonymous male in his 30s to reconstruct a bony model of the proximal femur using FEA software (MECHANICAL FINDER, Research Center for Computational Mechanics, Tokyo, Japan). This software provides a comprehensive range of tasks necessary for FEM, including the import of Digital Imaging and Communications in Medicine (DICOM) data, segmentation, mesh production, setting of various properties, and stress analysis of elements.

The CT equipment utilized in this study was the RHAPSODE system by GE Medical Systems. The imaging parameters used were as follows: 120 kVp, 120 mA, slice thickness of 3 mm, and pixel width of 0.546875 mm. To simplify the preparation process, tissues other than bony structures such as capsules, ligaments, or cartilage were excluded.

The data obtained from the CT scans were converted into DICOM format and transferred to generate the FE model [11]. The femoral trabecular bone and cortex were meshed into linear four-noded tetrahedral elements with a global edge length of 1 mm. This model did not include triangular shell elements that typically overlay the outer surface of these elements. The FE models consisted of 1,592,642 elements and 282,530 nodes, and the insufficient fracture line was represented with continuous void elements.

**Figure 1:**
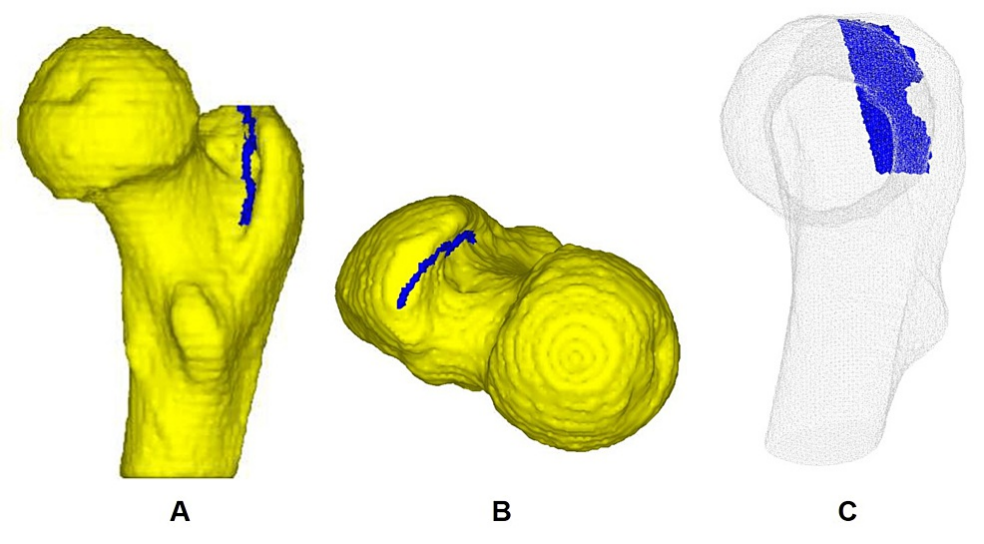
The femoral insufficient fracture model depicted in the fracture line. A: Posterior medial view, showing the fracture line (blue line) extending halfway down the intertrochanteric ridge. B: Superior view, displaying the fracture line running from the greater trochanter to the intertrochanteric ridge. C: Perspective view from the proximal femoral neck axis, illustrating the fracture line (blue plane) located posteriorly.

For this study, the following two models of the proximal femur were prepared: (1) a healthy proximal femur (HF) without fracture, and (2) a femoral insufficient intertrochanteric fracture (FIF) with a fracture line running from the tip of the greater trochanter posterolaterally along the intertrochanteric ridge to the middle of the femoral shaft, without creating a bony fragment at the greater trochanter, simulating critical fracture type for treatment strategy [6] (Figure 1).

Material properties

**Figure 2:**
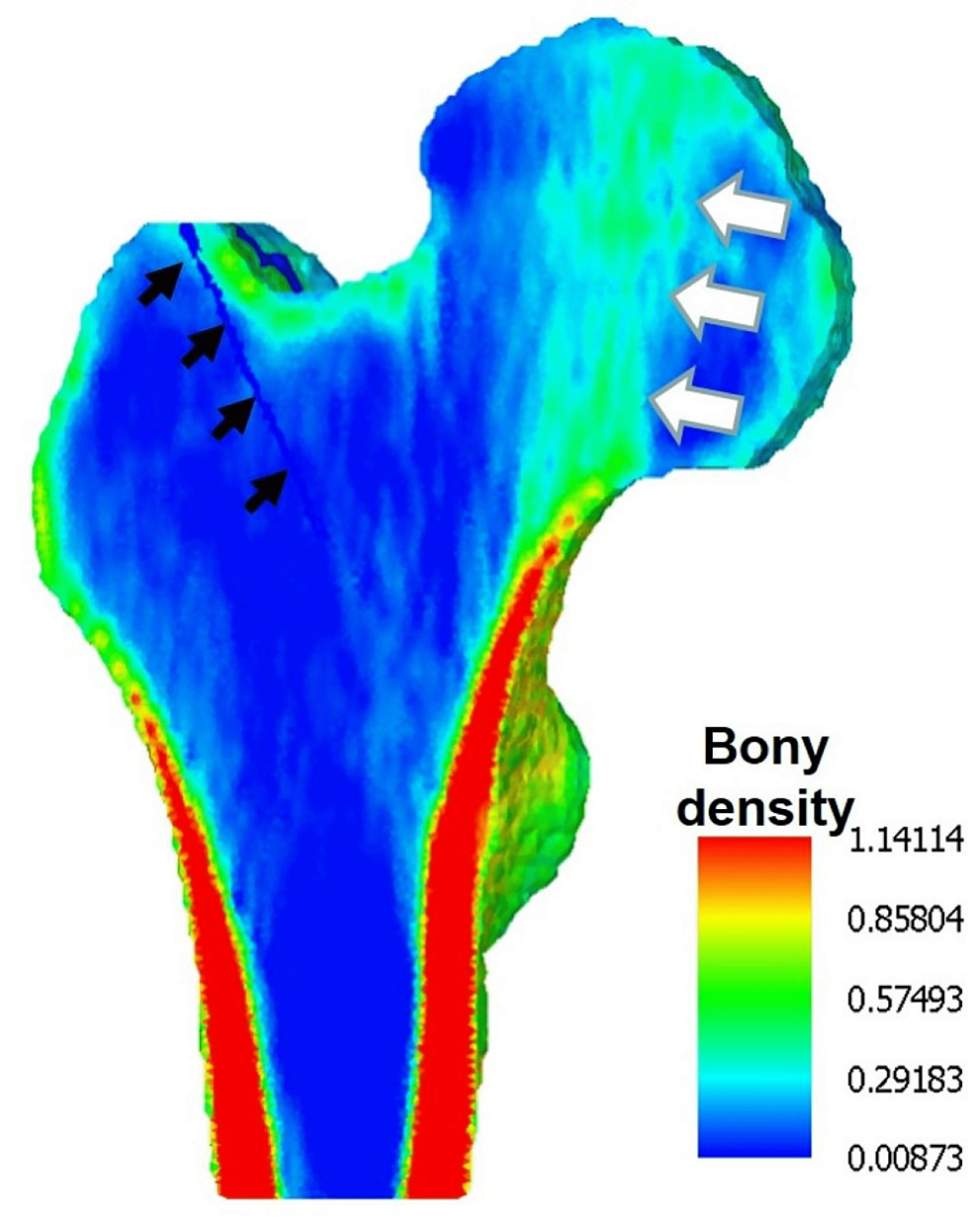
Map of bone density in a longitudinal sectional plane in an occult fracture model. The cortical bone at the femoral shaft, in line with the cervical neck, exhibits the highest bone density (depicted in red), with relatively higher density areas in the femoral head that support the head structure (indicated by white arrows), as well as the fracture line (depicted by black arrows). The color bar represents bone density (g/cm^3^).

The bone density (g/cm^3^) of osseous elements was estimated from the CT values using the following conversion formula, without using a phantom in this software (Figure 2): ρ (bone density) = (CT values + 1.4246) × 0.001/1.0580 [11].

The conversion from bone density to Young’s modulus (MPa) was performed using the equation proposed by Keyak et al. [12]. The bone density of the element representing the fracture line was set to 0 g/cm^3^ to reflect the Young’s modulus setting mentioned above. The yield stress was set according to the default setting in MECHANICAL FINDER, which assumes that elements with bone density less than 0.2 g/cm^3^ are elastic. A Poisson’s ratio of 0.3 was assigned to both the cortical and cancellous bone.

Loading conditions

Two types of loading forces were prepared, namely, (1) a simulated force for walking, coinciding with three times the body weight (3 W), and (b) touch-down standing with one-third of the body weight (1/3 W) [13]. For 3 W loading, a weight-bearing force of 1,500 N was applied to the hemispherical surface of the femoral head, and an abductor muscle force of 1,000 N was exerted on the insertion of the abductor musculature on the lateral and superior surfaces of the greater trochanter. For the 1/3 W loading, a weight-bearing force of 166.7 N was declined to the same spots on the femoral head, and an abductor muscle force of 111.1 N was added to the insertion of the abductor musculature. These forces were distributed among tens of elements to avoid causing exaggerated stress on each individual element.

Values of hip joint angles in the sagittal plane ranged from the neutral position (0° flexion) to midway (15° flexion) and the initial contact phase (23° flexion). These values were obtained from motion capture data during gait [14].

**Figure 3:**
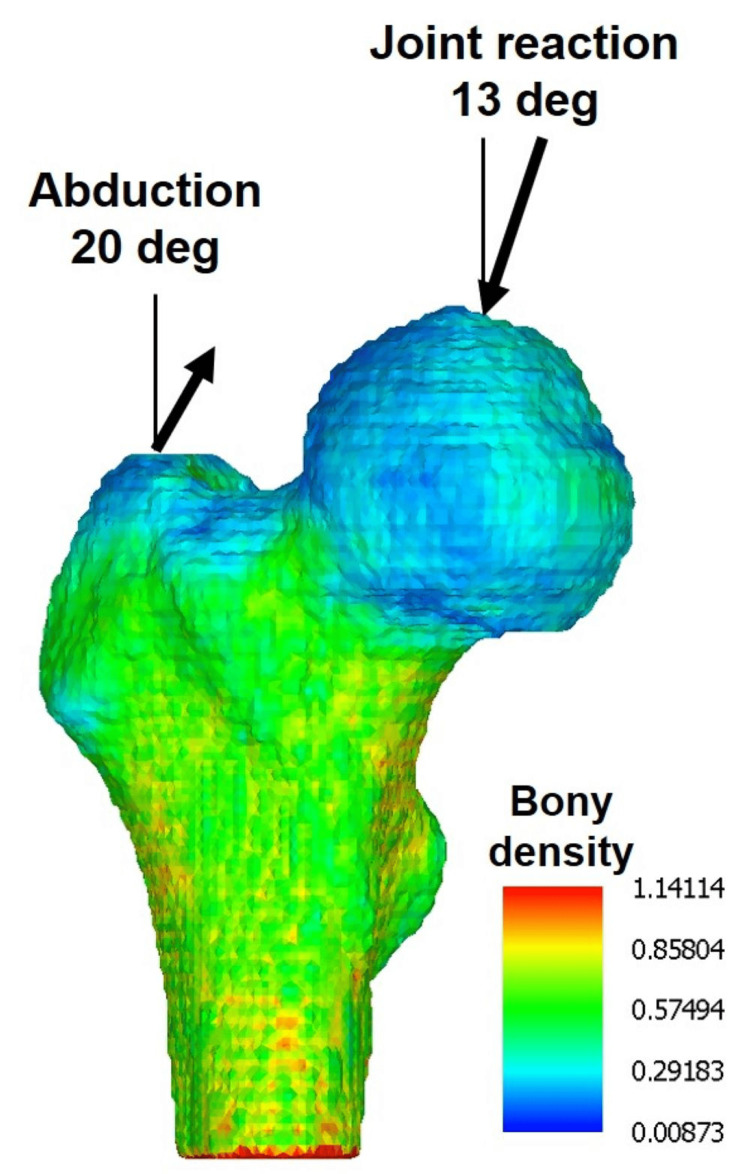
Direction of the external forces. The joint reaction force exerted on the femoral head is directed inward at a tilt of 13° in the coronal plane, while the abductor force acts on the tip of the greater trochanter, also tilted at 20°. The direction and magnitude of the muscle force remain constant. The color bar represents the bone density measured in g/cm³.

Regarding the loading angle in the coronary plane, the compression load at the top of the femoral head was inclined 13° from the vertical to the medial direction. Additionally, the abductor muscle force, acting as a tensile load, was tilted 20° from the vertical to the medial direction (Figure 3). The direction of the hip contact force and gluteus medius remained constant and formed a straight line. For hip flexion angles of 15° and 23°, instead of shifting the proximal femoral model against the ground, the load was tilted 15° and 23° forward in the sagittal plane, respectively. To evaluate the effects of different loading conditions, six combinations of two series of load magnitude (3 W, 1/3 W) and three different directions of loading (0°, 15°, and 23° flexion) were applied to each HF and FIF model.

As for the boundary conditions, the distal end of the femur was constrained in all directions (6 degrees of freedom).

Stress analysis using the finite element method

A stress analysis utilizing the FEM was performed employing the dynamic explicit approach. Von Mises stresses were calculated for the entire proximal femur to reveal the stress distribution in both HF and FIF models. The focus was placed on two designated spots on the ends of the insufficient fracture line (at the greater trochanter and the middle intertrochanteric ridge) to predict the risk of fracture extension. Additionally, the number of elements whose stress values exceeded the yield stress was counted to speculate whether the fracture would lead to a complete intertrochanteric fracture.

Ethical approval

Ethical approval was obtained for this study to use the provided CT graphical data from the Ethical Review Board of Nishi Hospital (approval number: 2022-1).

## Results

**Figure 4:**
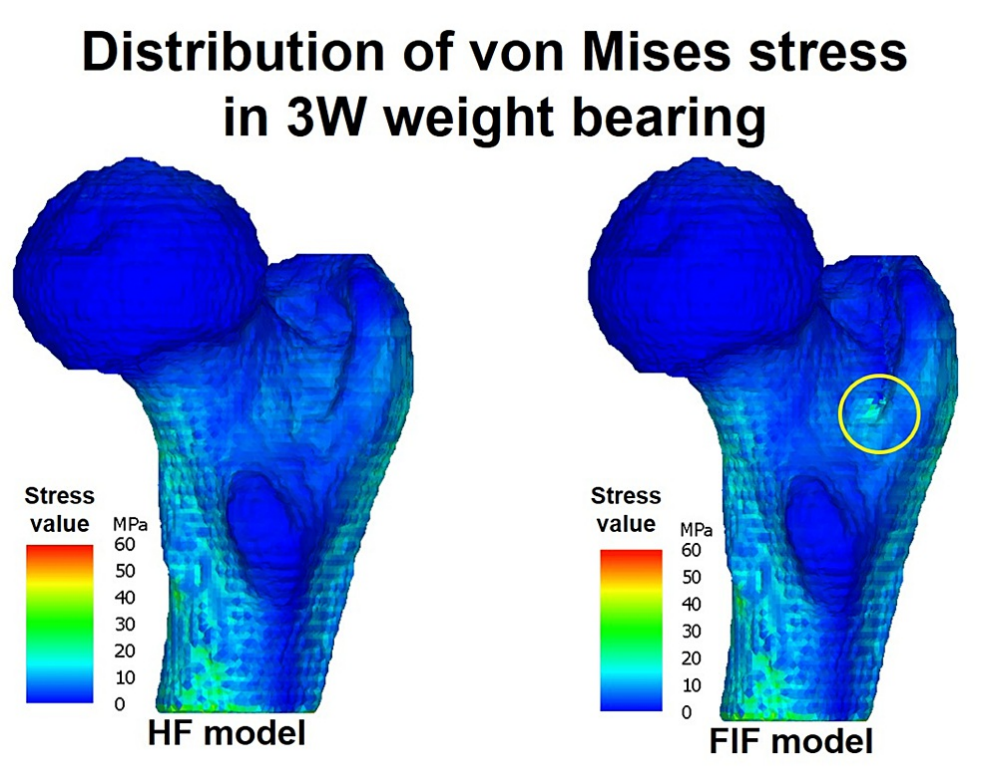
The stress distribution for walking (3 W). The stress distribution is simulated for walking (3 W) and observed from the posteromedial proximal femur at a hip joint flexion angle of 0 degrees in the HF model. It should be noted that the FIF model exhibits increased stress at the extension of the fracture line (marked by a circle) compared to the HF model. The color bar ranges from 0 to 60 MPa.

**Figure 5:**
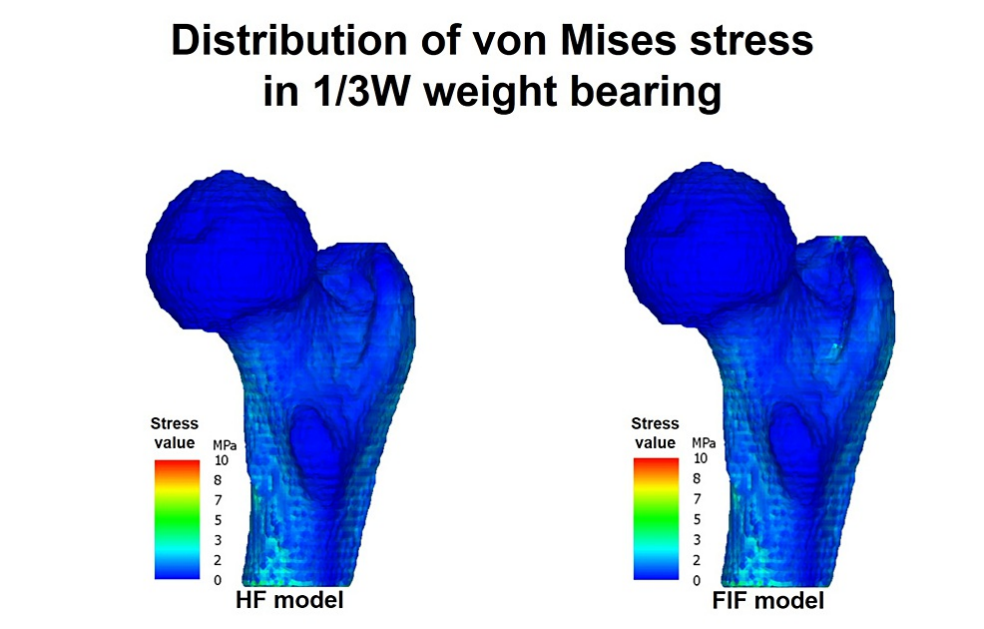
The stress distribution for 1/3 W. The stress distribution in the posteromedial proximal femur is simulated under partial weight-bearing conditions (1/3 W) with a hip joint flexion angle of 0°. The color bar illustrates the difference in stress values between the two models in Figure 4, ranging from 0 to 10 MPa.

**Figure 6:**
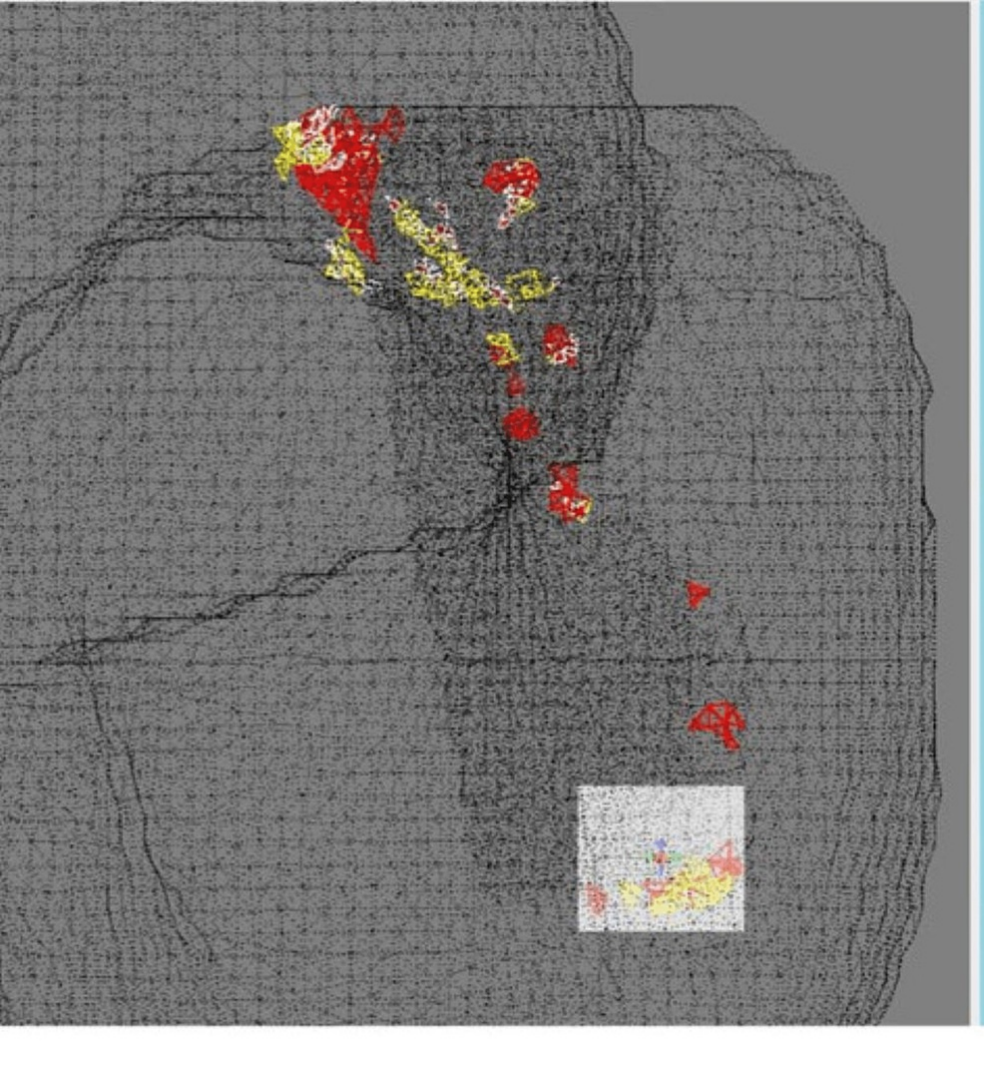
A perspective view of the occult fracture plane revealing elements exceeding the yield stress fracture line located posteriorly.

This study compared the distribution of von Mises stress between HF and FIF models in the entire proximal femur under the following six loading combinations: two weight-bearing conditions (3 W loading and 1/3 W standing) and three hip flexion angles (0°, 15°, and 23°). In the 3 W loading condition, there were no significant elevations in stress in the HF model at any flexion angle (Figure 4). However, the FIF model showed increased stress at the site of the posterior fracture line extension. In the 1/3 W loading condition, stress elevation was minimal in both HF and FIF models (Figure 5). In a plane parallel to the occult fracture plane, the stress substantially increased around the greater trochanter and the fracture line extension at the middle of the metaphysis (Figure 6). This destruction of elements was observed in both cortical and cancellous bone.

The number of yielded elements was summed along both edges of the fracture line (greater trochanter and middle of the intertrochanteric ridge) to assess the influence on fracture extension. In the 3 W loading condition, only one yielded element remained in the HF model, indicating little risk of fracture (Table 1). In contrast, the FIF model exhibited a notable presence of yield elements in various regions, indicated as total/greater trochanter/middle shaft, at different flexion angles as follows: 0° of 115/16/28, 15° of 265/158/23, and 23° of 446/233/34, respectively. In the 1/3 W loading condition, both HF and FIF models showed no yielding elements even with three directions of external force (Table 2).

**Table 1:** The number of elements exceeding yield stress around the fracture line in the walking (3 W) condition. The table presents the number of elements exceeding the yield stress threshold around the fracture line in the walking (3W) condition. The values are provided for two models at different hip angles (0°, 15°, and 23°). HF: healthy proximal femur; FIF: femoral insufficient intertrochanteric fracture

Hip flexion angle (°)	The designated spots at the end of the fracture line	HF model	FIF model
0	Total N	1	115
	Greater trochanter	0	16
	Middle shaft	0	28
15	Total N	0	265
	Greater trochanter	0	158
	Middle shaft	0	23
23	Total N	0	446
	Greater trochanter	0	233
	Middle shaft	0	34

**Table 2:** The number of elements exceeding yield stress around the fracture line in partial weight-bearing (1/3 W). The table presents the number of elements exceeding the yield stress threshold around the fracture line under partial weight-bearing conditions (1/3 W). The values are provided for two models at different hip angles (0°, 15°, and 23°). HF: healthy proximal femur; FIF: femoral insufficient intertrochanteric fracture

Hip flexion angle (°)	The designated spots at the end of the fracture line	HF model	FIF model
0	Total N	0	0
	Greater trochanter	0	0
	Middle shaft	0	0
15	Total N	0	0
	Greater trochanter	0	0
	Middle shaft	0	0
23	Total N	0	0
	Greater trochanter	0	0
	Middle shaft	0	0

## Discussion

The current FEM study consisted of the following two models: (1) a healthy femur without a fracture, and (2) an occult fracture with a fracture line extending up to 50% of the longitudinal axis on coronal images. The HF model showed minimal damage to the elements even under 3 W walking conditions. On the other hand, the FIF exhibited increased stress around the end elements of the fracture line located in the middle of the femoral shaft, surpassing the yield stress values. This fracture was further aggravated to induce medial extension of the intertrochanteric fracture and the fracture line, particularly in a flexed position [4]. However, when the weight-bearing condition was reduced to one-third of the body weight, the stress at the fracture site in the occult fracture model remained below the yield stress value, indicating a low risk of fracture extension.

Surgical indication

The surgical indication for this fracture has not been definitively established. Currently, the recommendation for operative intervention is primarily based on the degree of fracture extension observed on the coronal view [4,15]. The widely adopted protocol suggests that patients should undergo surgery when the fracture line reaches beyond 50% of the longitudinal axis on coronal images [4,6,15,16]. However, Oc et al. [17] advocated for surgical treatment for all patients, regardless of the spot of intertrochanteric fracture line extension on MRI, or the absence of medical contraindications [5]. The threshold value for conservative treatment shifted to a fracture line within the lateral two-thirds of the coronal plane, although the rehabilitation schedule varied depending on the site of fracture extension [18]. Another factor to consider is that a fracture angle between 35° and 42° reportedly does not require further imaging as it does not exclusively induce extension of the fracture line [16]. Holder et al. [19] assumed the presence of a pacemaker as a key consideration favoring surgical intervention due to the prohibition of MRI use. Based on our study, it is important to consider not only the extent of the fracture line but also the patient’s compliance with the rehabilitation protocol before making a decision between surgical and conservative treatment.

Magnetic resonance imaging or computed tomography study of this fracture

MRI or CT scan is widely recognized as a useful tool for diagnosing occult hip fractures [20]. MRI is particularly effective in detecting occult fractures and is routinely recommended as the primary imaging modality [5]. A systematic review and meta-analysis of MRI protocols for detecting radiographically occult proximal femoral fractures demonstrated excellent results [21]. The study reported sensitivity and specificity values of nearly 100% when using T1-weighted and/or short tau inversion recovery sequences. However, MRI has its limitations, as many hospitals may not have easy access to this imaging modality. Additionally, MRI tends to over-interpret findings by highlighting the higher sensitivity of bone marrow edema, soft tissue abnormalities, and cancellous bone pathology [22]. Therefore, although MRI may be efficient for accurate diagnosis, surgeons should exercise caution as the extent of the fracture line may be exaggerated when determining the border of the fracture line. In other words, even if the fracture line appears to exceed well over the half diameter of the femur on MRI, the real fracture line may remain within the lateral half.

High-quality CT is a valuable tool for assessing bony structural morphology. However, it may not always effectively detect nondisplaced hip fractures [4,20,23]. Compared to ordinary plain radiography, CT or three-dimensional CT has higher sensitivity but lower sensitivity than MRI [24].

Conservative treatment

Conservative management of occult intertrochanteric fractures has shown satisfactory outcomes with various immobilization protocols. Typically, patients undergo bed rest for one to three weeks, followed by progressive walker-assisted ambulation [15]. The initiation of a walker or crutches is determined by pain relief [15,24]. In some patients, implementing exercise is delayed for a period of one month, during which they exercise in bed, and subsequently commence walking without assistive devices after a two-month period [25]. So far, it has not been proven that such a prolonged rehabilitation affects end functional results unfavorably. Chinzei et al. [26] compared early versus delayed surgery in bipolar arthroplasty and found no significant differences in muscular strength and other functional indices for at least 1.5 years postoperatively. Surgeons who encourage surgical intervention for all occult fractures, fearing delayed surgery, should consider a decline in functional outcomes associated with delayed operations.

Strengths and limitations

This study has several strengths. First, it quantifies the risk of re-fracture by identifying the number of elements susceptible to yield stress. Second, it is grounded in scientific background, in contrast to previous clinical articles lacking clear bases. Third, this research adopts a clinical viewpoint, considering various load and joint flexion angles, which contributes to its significance in clinical practice. Fourth, the refined model incorporates heterogeneous material properties by converting CT values, including Young’s modulus, without assuming a two-layer model of homogeneous material properties [14].

However, this study also has its limitations. First, the model lacks the inclusion of soft tissues such as the joint capsule, ligaments, and muscles. Second, as the current study only simulates static loading, it may not accurately reproduce situations encountered in dynamic daily life [14]. Third, the femoral head is not constrained against the acetabulum, resulting in the absence of physiological behavior around the femoral head. Fourth, our model was derived from CT graphical data of a young, healthy male, whereas most occult fractures occur in aged, osteoporotic females.

Future directions

Although the present study revealed that partial weight-bearing and restricted flexion angle facilitate conservative management, we believe that surgical indications should also depend on multiple factors, such as bone density, inclination of the fracture line, and the size of the greater trochanteric fragment. Therefore, we are planning further investigations to explore the combined effects of the factors mentioned above, as well as individualized properties, including the use of more osteoporotic materials.

Surgeons should also focus not only on achieving bony union exclusively but also on functional recovery among surgically and conservatively treated groups. Ren et al. [25] pointed out that conservative treatment of an isolated greater trochanteric fracture attenuates the abductive strength of the hip joint (137.4 N on the healthy side versus 121.4 N on the injured side) in a short-term follow-up, which they argued is related to injuries of the ligaments and muscles around the greater trochanter. In contrast, our preliminary study demonstrated a more significant decrease in abduction strength among patients treated with gamma nailing, primarily due to muscular damage caused by nail drilling [27].

## Conclusions

The current FEM study provided two sets of models, namely, a healthy model without a fracture, and an occult fracture model with a fracture line extending up to the mid-shaft on coronal images, under various combined conditions of different weight-bearing forces and hip joint angles. The healthy model exhibited minimal damage to the elements under all walking conditions. In the occult fracture model, during walking (3 W), stress levels increased above the yield stress values at the end elements of the fracture line located in the middle of the femoral shaft. This was more pronounced in the flexed position. When the weight-bearing condition was reduced to one-third of the body weight, the stress at the fracture site remained within the range of the yield stress value, indicating a relatively low risk of fracture extension. The clinical implications of this study suggest that the surgical protocol should take into account patients’ compliance with weight-bearing restrictions in this type of fracture.
